# Real-world federated learning for brain imaging scientists

**DOI:** 10.3389/fdgth.2026.1691088

**Published:** 2026-03-13

**Authors:** Stijn Denissen, Jorne Laton, Matthias Grothe, Manuela Vaneckova, Tomáš Uher, Matěj Kudrna, Dana Horáková, Johan Baijot, Iris-Katharina Penner, Michael Kirsch, Jiří Motýl, Maarten De Vos, Oliver Y. Chén, Jeroen Van Schependom, Diana Maria Sima, Guy Nagels

**Affiliations:** 1 AIMS Lab, Center for Neurosciences, UZ Brussel, Vrije Universiteit Brussel, Brussels, Belgium; 2 Department of Radiology, First Faculty of Medicine, Charles University, General University Hospital, Prague, Czechia; 3 icometrix, Leuven, Belgium; 4 Department of Neurology, University Medicine Greifswald, Greifswald, Germany; 5 Department of Neurology and Center of Clinical Neuroscience, First Faculty of Medicine, Charles University, General University Hospital, Prague, Czechia; 6 Department of Neurology, Inselspital, Bern University Hospital, University of Bern, Bern, Switzerland; 7 Institute for Diagnostic Radiology and Neuroradiology, University Medicine of Greifswald, Greifswald, Germany; 8 Department of Electrical Engineering (ESAT), KU Leuven, Leuven, Belgium; 9 Development and Regeneration, KU Leuven, Leuven, Belgium; 10 Département Médecine de Laboratoire et Pathologie (DMLP), Centre Hospitalier Universitaire Vaudois (CHUV), Lausanne, Switzerland; 11 Faculté de Biologie et de Médecine (FBM), Université de Lausanne, Lausanne, Switzerland; 12 Department of Electronics and Informatics (ETRO), Vrije Universiteit Brussel, Brussels, Belgium; 13 St Edmund Hall, University of Oxford, Oxford, United Kingdom

**Keywords:** BIDS, brain, brain age, cognition, deep learning, federated learning, multiple sclerosis

## Abstract

**Background:**

Federated learning (FL) has the potential to boost deep learning in neuroimaging but is rarely deployed in real-world scenarios, where its true potential lies. We propose FLightcase, a new FL toolbox tailored for brain research, and evaluate it on a real-world FL network to predict the cognitive status in patients with multiple sclerosis (MS) from brain magnetic resonance imaging (MRI).

**Methods:**

We first trained a DenseNet neural network to predict age from T1-weighted brain MRI on three open-source datasets: IXI (586 images), SALD (491 images), and CamCAN (653 images). These were distributed across the three centres in our FL network: Brussels (BE), Greifswald (DE), and Prague (CZ). We benchmarked this federated model with a centralised version. The best-performing brain age model was then fine-tuned to predict performance on the symbol digit modalities test (SDMT) of patients with MS (Brussels: 96 images, Greifswald: 756 images, Prague: 2,424 images). Shallow transfer learning (TL) was compared with deep transfer learning, in which weights were updated either in the last layer or across the entire network, respectively.

**Results:**

Federated training outperformed centralised training, predicting age with a mean absolute error (MAE) of 6.08 versus 7.02. Federated training yielded Pearson correlations (all *p* < .001) between true and predicted age of0.88 (IXI, Brussels), 0.91 (SALD, Greifswald), and 0.93 (CamCAN, Prague). Fine-tuning of the centralised model to SDMT was most successful with a deep TL paradigm (MAE = 9.19) compared to shallow TL (MAE = 11.05). Across Brussels, Greifswald, and Prague, deep TL predicted SDMT with MAEs of 10.71, 9.67, and 8.98, respectively, and yielded Pearson correlations between true and predicted SDMT of.25 (*p* = 0.282), 0.40 (*p* < 0.001), and 0.50 (*p* < 0.001).

**Conclusion:**

Real-world federated learning using FLightcase is feasible for neuroimaging research in MS, enabling access to large MS imaging databases without sharing data. The federated SDMT-decoding model is promising and could be improved in the future by adopting FL algorithms that address the non-IID data issue and consider other imaging modalities. We hope our detailed real-world experiments and open-source distribution of FLightcase will prompt researchers to move beyond simulated FL environments.

## Introduction

Deep learning is gaining traction as a tool to study the brain's function and structure (1, 2). Since the comprehensive Nature review by Lecun et al. (3), interest among brain researchers has surged; more than 10,000 papers have been published on the topic in the past decade, compared just over 600 by the end of 2014.

Deep learning has led to major breakthroughs in brain analysis. Brain structures can now be accurately quantified using segmentation models (4, 5), aiding doctors in diagnosis and treatment evaluation. Deep neural networks also reduce the error in predicting age from brain magnetic resonance imaging (MRI) to the order of 2 years (6). This yields accurate biological aging clocks that capture deviations from healthy aging patterns and serve as efficient communication tools, allowing brain damage to be expressed in terms of “how much older the brain looks.” Deep learning models can indeed capture the brain's complexity by creating high-dimensional, data-driven representations beyond human understanding. However, there is a catch. To create reliable representations, deep learning models require big training datasets, typically in the order of tens of thousands of images (6). As many dedicated researchers have invested significant effort in collecting datasets, we need to reconsider how they can be reused and combined to unlock the full potential of deep learning in brain image analysis.

The conventional way of training deep learning models involves centralising datasets. This is feasible for certain domains like brain age, as it relies on healthy control datasets that are publicly shared. Indeed, age-labelled T1-weighted images are widely available via initiatives such as the UK Biobank (7) and many repositories in OpenNeuro (8). In most other domains, however, particularly those working with sensitive patient data, data sharing is difficult. Barriers to data sharing are numerous, spanning in technical, motivational, economic, political, legal, and ethical terms (9). The latter three domains are especially relevant for centralising neuroimaging data. However, the first condition is trust: “In the absence of trust, providers could anticipate potential misinterpretation, misuse or intentional abuse of the data” (9). Second, the global data protection regulation (GDPR) enforces strict guidelines on data sharing. This can complicate the procedure, lower incentives, or block sharing entirely. This is especially true for the type of data that brain scientists work with, as faces can be reconstructed from MR images. Lastly, data sharing is relatively static. Data from routine clinical practice is continuously generated, and sharing those periodically is time-consuming and inefficient.

This conventional, centralised view on machine learning was challenged by McMahan et al. (10). They introduced the concept of federated learning (FL), in which models are trained at local institutions. Instead of sharing data, models are shared. Moreover, the computational load and data storage are spread across multiple centres. In the following years, brain researchers started experimenting with FL in simulated settings, primarily using open-source data. New algorithms were proposed (11), performance with respect to centralised training was explored (12), and federated learning toolboxes were designed (13). While FL in principle solves all previously mentioned issues, it generates new challenges that hinder brain scientists from deploy it in a real-world setting. These include financial constraints [e.g., graphical processing units (GPUs)], hardware and software differences, connectivity issues, and data heterogeneity. To date, only a few groups have succeeded, mainly in brain tumour segmentation (14, 15), where access to bigger datasets boosted model performance (15). While other real-world examples exist, it is often unclear from published methods whether they involved a simulation or real-world FL on geographically distributed data.

In this work, we aimed to pioneer real-world FL in our modelling domain, decoding cognitive performance from structural brain MRI in patients with multiple sclerosis (MS), using data from three international centres in Brussels (BE), Greifswald (DE), and Prague (CZ). When initiating the practical setup in early 2023, the most pressing challenge was the lack of software capable of orchestrating real-world federated learning in our neuroimaging context and of handling clinical datasets that differed widely in format. Therefore, we designed a simple, glass-box FL framework that allows easy real-world deployment in an international context: “FLightcase.” FLightcase is designed to work with the Brain Imaging Data Structure [BIDS (16)], which has become the standard data organisational format for brain imaging data over the past decade (17). BIDS is enforced by data sharing platforms such as OpenNeuro (8), and we sought to continue this trend. By doing so, we aim to encourage brain AI researchers to adopt decentralised model training and leverage larger, multi-institutional datasets to boost generalisability.

The contributions of this paper are as follows:

- We introduce FLightcase, a simple, open-source, BIDS-compliant FL toolbox for neuroimaging.
- We demonstrate the real-world readiness of FLightcase on a real-world FL network with geographically distributed data from Brussels (BE), Greifswald (DE), and Prague (CZ). The modelling goal was to predict cognitive function from brain MRI in MS using transfer learning (TL) from a pre-trained brain age model. We believe this is possible as cognitive deterioration naturally occurs with aging (18) and as we previously found a link between brain age and cognition in MS (19). If successful, the model could be employed as a cognitive screening tool on routine brain imaging, which is recommended at least yearly in people with MS (20). In the real-world experiments, we addressed two research questions:
  – Does federated training match centralised training in terms of model performance? This question was addressed in brain age modelling, as it relies on open-source data that can be centralised.
  – Does deep transfer learning (updating all network weights) outperform shallow transfer learning (updating only the weights of the fully connected layer) for predicting cognitive impairment in MS?
- We discuss challenges and solutions in setting up a real-world federated learning network to encourage the method in the field.

## Methods

### FLightcase in brief

FLightcase is a federated learning toolbox that was specifically designed to work with the brain imaging data structure (BIDS) (16). We aim to facilitate model training for brain researchers and stimulate researchers to use this data structure. Its basic communication relies on sending two files sequentially. The first file contains the machine learning information to be transmitted and the second is a text file marking transmission completion. Files are sent between computers via secure copy protocol (SCP), which requires all participating computers to be UNIX-based. In this paper, the terms “computer” and “node” are used interchangeably. The SCP command requires two localisers:

- The IP address of the receiving computer is required. An example of a secure network where computers are assigned an IP address, and are therefore reachable, is a virtual private network (VPN). This was used in our real-world example (cfr. infra).
- The receiver location for the file within the computer is also necessary. To facilitate this, we use the concept of an “FL workspace.” The FL workspace is a folder dedicated to the federated learning experiment.

These and other settings are stored in a JavaScript Object Notation (JSON) file per computer. They are different for server and clients, for which templates can be found in Supplementary Table S1 and S2 respectively. For example, the client settings define the location of the BIDS dataset, while the server settings define the expected clients. To orchestrate the FL process, the server moreover stores an “FL plan” JSON file containing training preferences (e.g., number of FL rounds). Lastly, the server defines the model architecture that will be trained in a separate Python file.

For a detailed overview of the FLightcase software, refer to the “FLightcase unpacked” section of the Supplementary Material. FLightcase is publicly available in our AIMS-VUB GitHub repository (https://github.com/AIMS-VUB/FLightcase, branch “FL_POC”) and is deployed on the Python Package Index (PyPI, https://pypi.org) for easy installation. The federated experiments in this study used FLightcase v0.1.17. FLightcase can be tested using the simulation provided in the Supplementary Material, section “How to test FLightcase.”

FLightcase v0.1.17 depends on the following Python modules: torch v2.5.1 (21), pandas v2.2.3 (22), monai v1.4.0 (23), scikit-learn v1.6.0 (24), tqdm v4.67.1 (25), nibabel v5.3.2 (26), paramiko v3.5.0 (27), scp v0.15.0 (28), matplotlib v3.10.0 (29), scipy v1.14.0 (30), numpy v1.26.4 (31), click v8.1.8 (32), and twine v6.0.1 (33).

### The federated learning network

**Figure 1:**
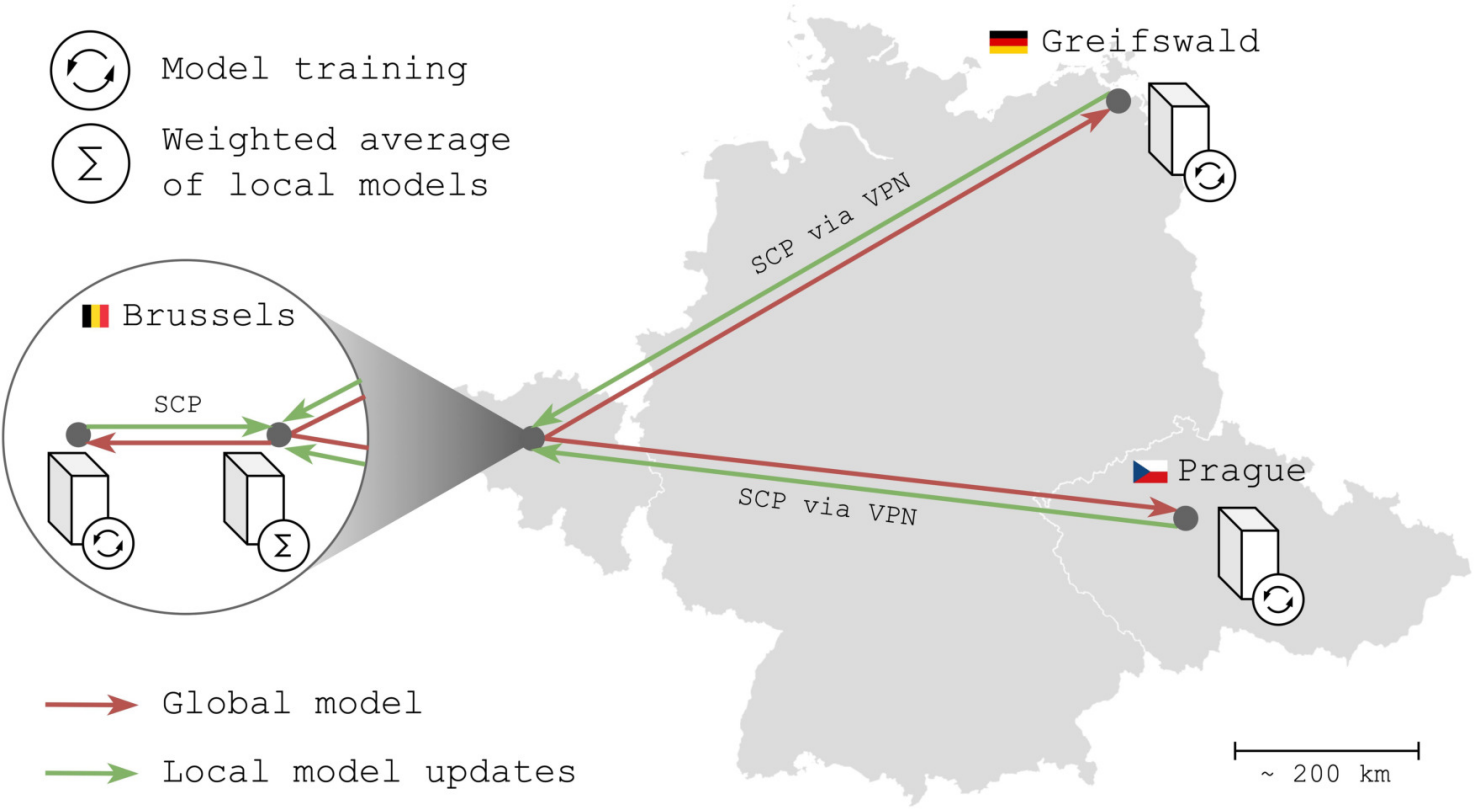
The federated learning network for a real-world experiment. The computer with the “sigma” symbol is the server, whereas computers with an “update” symbol are clients. SCP, secure copy protocol; VPN, virtual private network. Belgium, Germany and Czech Republic flags from flagcolorcodes (https://www.flagcolorcodes.com/). Icons from JGraph (https://github.com/jgraph), licensed under CC BY 4.0

To prove the real-world effectiveness of FLightcase, we tested it in an FL network across our AIMS labs in Brussels (BE), the Greifswald University Hospital (DE), and the General University Hospital Prague (CZ). The network (Figure 1) consisted of four computers, of which one is the server that coordinates the project and the other three serve as clients on which models are trained using the local data. The two Brussels computers were located in the same office and connected to the network of the Department of Electronics and Informatics (ETRO) at VUB. The computers in Greifswald and Prague were connected to this network via a VPN. Models were shared via secure copy protocol (SCP) with secure shell (SSH).

All client computers were equipped with graphical processing units (GPUs): Brussels: NVIDIA GeForce RTX 4090 (24GB), Greifswald: NVIDIA GeForce RTX 3090 (24GB); and Prague: NVIDIA GeForce RTX 4090 (24GB). The operating system of each computer (server and clients) was Debian GNU/Linux 12 (“bookworm”), and Python version 3.11.2 was used consistently.

### Real-world FLightcase testing

Testing FLightcase on the real-world FL network involved two steps. Step 1 involved training a DenseNet convolutional network (34) to predict age from T1-weighted brain MRI, similar to the work of James Wood and colleagues (35). As step 1 was modelled on open-source data and thus allowed centralising of data, federated training was benchmarked against centralised training (explained at the end of the Methods section). Step 2 involved transfer learning of the best brain age model from step 1 [lowest overall test mean absolute error (MAE)] to predict cognitive impairment in people with MS.

The FL plans containing the hyperparameters for federated training are provided in Supplementary Table 4. We used a batch size of 10. Federated averaging (FedAvg) was used consistently, given its popularity in the field (36, 37) and in line with our emphasis on simplicity to enable real-world FL. At the end of each federated learning round, the FL server selected a sample of two out of three client models and performed a weighted average across both. The weight of each client model was defined by the sample size of that client divided by the total sample size of both clients in the sample.

### Ethics

The “Commissie Medische Ethiek” (CME) of UZ Brussel judged this retrospective study to be exempt from ethical approval (B.U.N. 1432022000303). For the MS data at each centre in this study, ethical approval was obtained prior to data acquisition (Brussels: B.U.N. 143201423263, Greifswald: BB159/18, Prague: 113/22 S-IV and 28/17). Healthy control data used to train the brain age model was obtained from open-source datasets.

### Data

To train the brain age network (step 1), we used three open-source datasets: the Cambridge Centre for Ageing and Neuroscience (CamCAN) dataset (38), the Information eXtraction from Images (IXI) dataset (39), and the Southwest University Adult Lifespan Dataset (SALD) (40). The CamCAN dataset was stored on the Prague computer, the IXI dataset in Brussels and the SALD dataset in Greifswald. The datasets contained T1-weighted MRI, sex at assessment, and age from healthy subjects. The data are summarised in Table 1.

**Table 1:** Dataset characteristics.

Variable	Open-source (step 1)			Closed-source (step 2)		
	CamCAN	IXI	SALD	Brussels	Greifswald	Prague
*N*	653	586	491	96	338	916
*N* image—SDMT pairs	653	586	491	96	756	2,424
Sex at assessment (m:f)	323:330	258:328	185:306	27:69	110:228	275:641
Age at scan (*M* ± SD)	54.3 ± 18.6	49.4 ± 16.7	45.2 ± 17.5	47.9 ± 9.9	43.5 ± 12.1	42.2 ± 9.5
SDMT (*M* ± SD)	/	/	/	48.0 ± 11.6	51.7 ± 15.3	58.9 ± 12.0
EDSS (Med; IQR)	/	/	/	3; 2	2; 2	2.5; 2
Disease duration (*M* ± SD)	/	/	/	15.5 ± 8.5	9.2 ± 6.9	13.2 ± 8.1
Type MS	/	/	/	CIS: 2 RRMS: 81, SPMS: 6, PPMS: 7	CIS: 7 RRMS: 680, SPMS: 41, PPMS: 23, RIS: 5	CIS: 448, RRMS: 1566, SPMS: 363, PPMS: 38, PRMS: 6
T1w MR images						
Manufacturer	Siemens	Philips, GE	Siemens	Philips	Siemens	Siemens
Model	TrioTim	Philips: Intera, Gyroscan Intera	TrioTim	10. Achieva: 31 Ingenia: 65	Verio	Skyra
Echo time (ms)	2.98	4.603	2.52	2.303; 2.287	2.58	2.96
Repetition time (ms)	2.25	9.600; 9.813	1.9	4.952; 5.189	1.900	2.300
Slice thickness (mm)	1	/	/	1.0	0.90	1
Flip angle (degrees)	9	8	9	8	9	9
Field strength (*T*)	3	3 and 1.5	3	3	3	3

*n*, sample size; m, male; f, female; *M*, mean; SD, standard deviation; SDMT, symbol digit modalities test; EDSS, expanded disability status scale; GE, general electric. (1) Variable distributions are calculated across all image-SDMT pairs. (2) Missing values: 14 EDSS (Prague), 3 disease course (Prague), and 15 disease duration (Greifswald). (3) The MR image acquisition info was extracted from a single subject per scanner model and might therefore deviate among subjects. For IXI, which was acquired at three different sites, MRI acquisition info was obtained from the website: https://brain-development.org/ixi-dataset/ (accessed March 10, 2025). Detailed scanner info was only available for the Philips scanners.

The cognition prediction network (step 2) was trained on three MS datasets at the Vrije Universiteit Brussel (VUB), the General University Hospital Prague (VFN), and Greifswald University Hospital. The data were organised locally in the BIDS format and contained T1-weighted MR images, demographics, and clinical information. This contained sex at assessment, age, expanded disability status scale [EDSS (41), physical disability], disease duration, MS subtype (relapsing versus progressive onset), and the symbol digit modalities test [SDMT (42)], i.e., the target to predict. We chose the SDMT as it is the most sensitive to cognitive impairment in MS (42, 43) and as we previously found a link between brain age and SDMT in people with MS (19). The popularity of the SDMT in MS research and care is reflected in its presence in all modern cognitive test batteries (42). As a result, choosing the SDMT enabled us to extract the largest possible decentralised cognition-labelled MRI database. In the SDMT, a subject is presented a list of symbols that need to be converted to numbers using a key at the top of the page, matching symbols with numbers. In 90 s, the subject must convert as many symbols to numbers as possible, each time saying the number out loud for the test administrator to write down. The SDMT is a measure of information processing speed.

The T1-weighted MR images were pre-processed using the pipeline described by Wood et al. (35). The pipeline first aligns a volume to the right anterior superior (RAS) orientation, and then applies skull-stripping using the HD-BET brain extraction algorithm (44). The image is then bias field-corrected using the N4 algorithm from Advanced Normalisation Tools [ANTs, (45)]. Also using ANTs, the image is affine registered to Montreal Neurosciences Institute (MNI) 152 space (1 mm isotropic) using all 12 degrees of freedom (rotation, translation, scaling, and shearing). After ensuring that the volume is in RAS orientation, the image is resampled with a voxel spacing of 1.4 and cropped/zero-padded to a window of 130 × 130 × 130 voxels. All steps are defined in the “pre_process.py” script (https://github.com/MIDIconsortium/BrainAge/blob/main/pre_process.py). An updated version of this script was included in our GitHub repository with permission from the authors (35).

The data were randomly split into 80% train and 20% test data on each client. To prevent data leakage, multiple images of a single subject were collected in either the training or test set. During each FL round, the training data were bootstrapped five times, with 75% for training and 25% for validation. This resulted in a train/validation/test split of 60/20/20 (cfr. Supplementary Table 4).

#### The 3D DenseNet model

**Figure 2:**
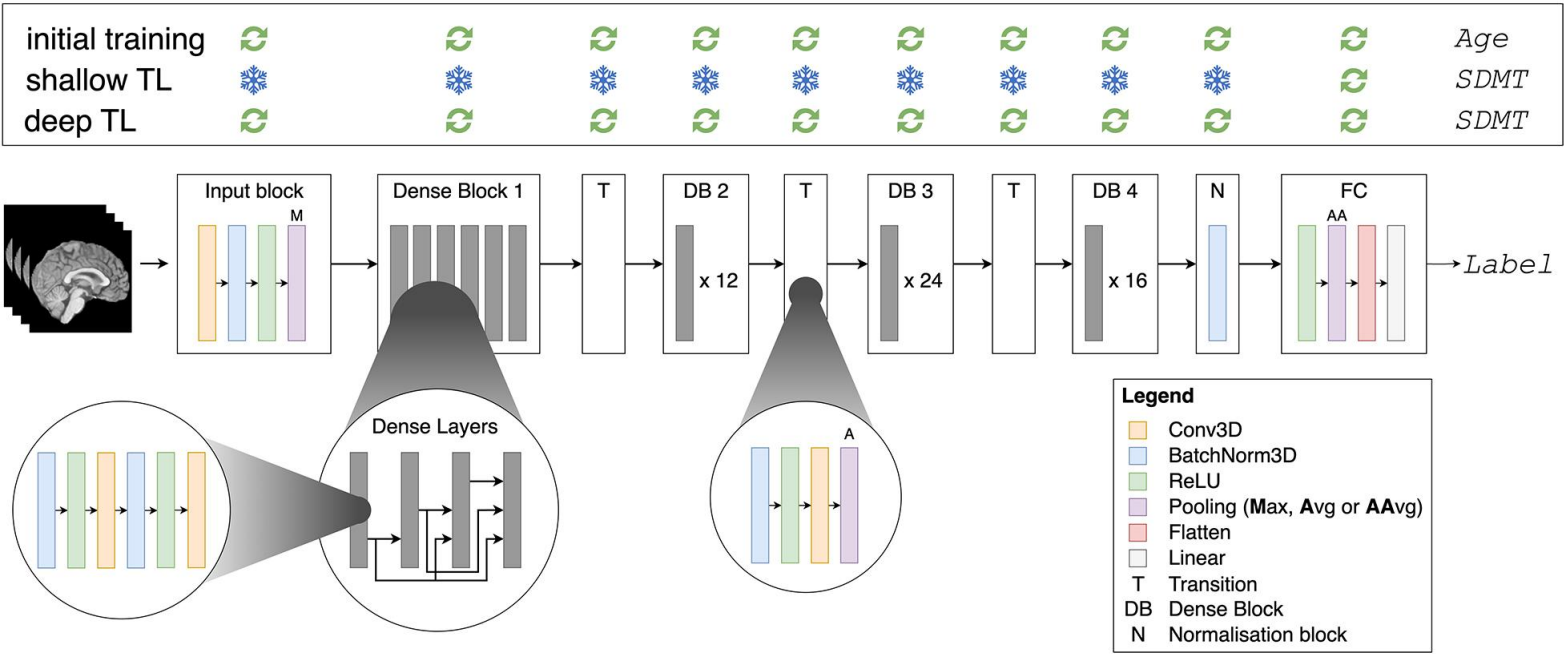
Densenet architecture and training methodology (top panel). Avg, average; AAvg, adaptive average. The “Label” is the chronological age for the initial brain age prediction task, and the SDMT for the shallow and deep transfer learning (TL).

The 3D Dense Convolutional Network (DenseNet) (34) was used during both the initial brain age task (step 1) and the transfer learning task to SDMT (step 2). DenseNet has outperformed other network architectures in a medical imaging context (46) and is unique in directly connecting all layers inside the network with each other (34). Each layer therefore takes all previous feature maps—outputs of previous layers—as input, improving the propagation of features throughout the network. The DenseNet architecture moreover reduces the “vanishing gradient” problem, where gradients used to update weights in the network gradually approach zero during backpropagation to earlier layers. Lastly, the model reduces the number of parameters in the network (34). The 3D DenseNet model in this paper has 11,243,649 updatable parameters and is schematically illustrated in Figure 2.

For the brain age prediction task, all layers in the network were unfrozen, whereas for the SDMT prediction task, two transfer learning methods were explored. In the “shallow TL” task, only the fully connected layer, consisting of 1,025 parameters (1,024 weights and one bias), was updated during training (cfr. Figure 2); the other layers—the feature extractor parts of the network—were frozen. In the “deep TL” task, all layers of the network were updated. Figure 2 also summarises the transfer learning methodology in the box on top.

#### Evaluation

The final performance of all models was evaluated on the test dataset per client using the MAE and Pearson correlation. Overall model performance was calculated using Equation 1:

$$\mathrm{MA}E_{\mathrm{test},\mathrm{overall}}=\sum_{i\;=\;0}^{m}\frac{\mathrm{MA}E_{\mathrm{test},i}*n_{i}}{N}$$

Equation 1 Overall test MAE calculation, where m is the number of clients and *N* is the total sample size across clients.

Lastly, for the brain age analyses, we also reported the Pearson correlation between the brain age difference [BAD, predicted age (brain age) minus the calendar age] and calendar age to investigate a potential bias (47).

### Benchmarking: centralised brain age training

As a benchmark model, we additionally updated the brain age model on a centralised version of the three open-source datasets. Here, we mimicked federated training as closely as possible by syncing the hyperparameters with the federated experiment (Supplementary Table 4), aggregating the test datasets to a centralised version, and using an aggregated version of the same train/validation splits used in federated training. As the centralised setting had access to all test results together, the test MAE was calculated across all subjects simultaneously instead of the per-client approach in Equation 1.

## Results

### Step 1: predicting brain age

#### Real-world federated training

**Figure 3:**
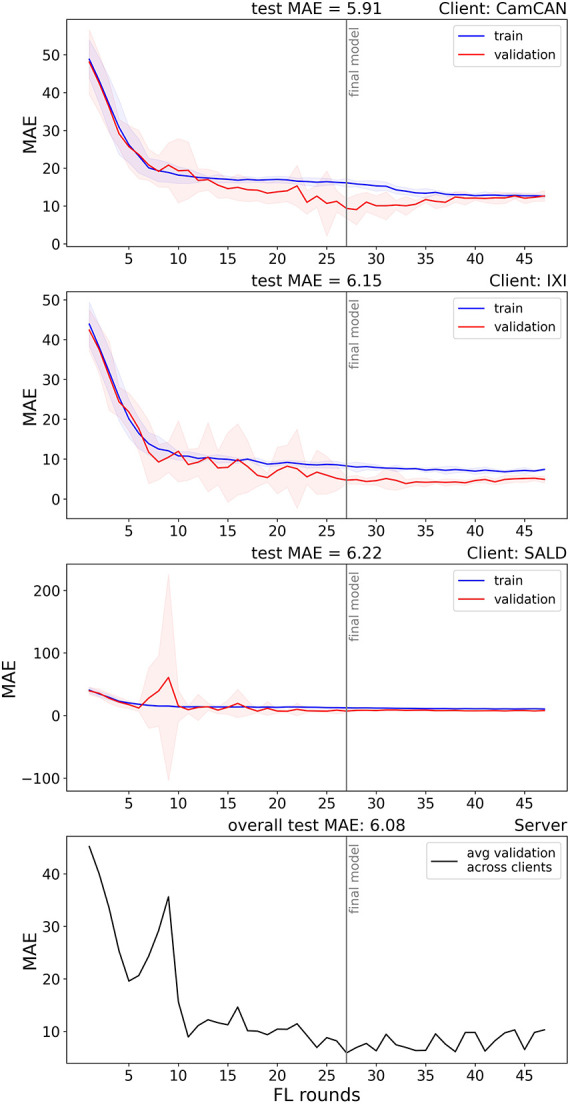
Federated training of the brain age network. The red (train) and blue (validation) lines represent the average train and validation MAE, respectively, and the shaded red and blue areas represent the 95% confidence interval, all calculated across five bootstraps per FL round. The right lower panel shows the average validation MAE across the clients in the aggregation, calculated on the server side.

**Figure 4:**
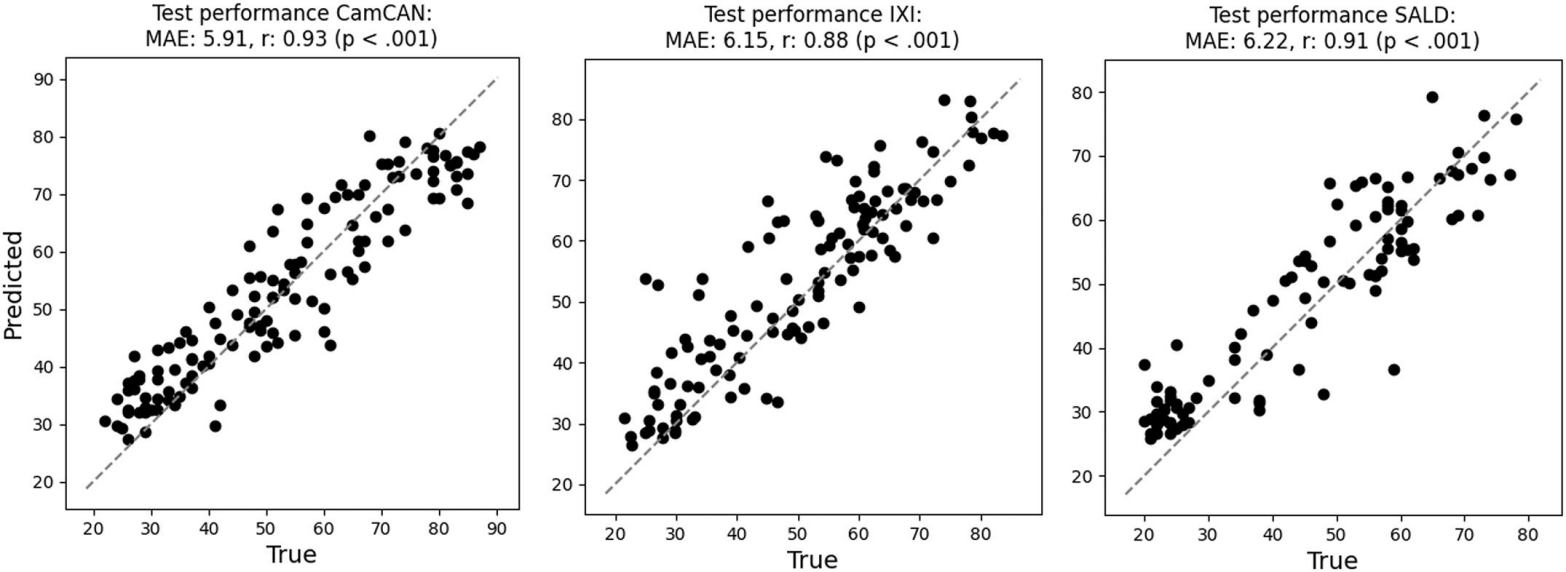
Scatterplots between true and predicted age (brain age) on the test datasets of CamCAN (stored in Prague), IXI (stored in Brussels), and SALD (stored in Greifswald). The grey dashed line indicates the perfect prediction line where age equals brain age.

The total FL process took 1 h, 48 min, and 21 s to complete. The model reached a minimum after 48 rounds and training stopped early after 27 rounds. The final network achieved an overall test MAE of 6.08, and an MAE of 5.91, 6.15, and 6.22 on the test datasets of CamCAN, IXI, and SALD, respectively. Pearson correlations for the respective datasets were 0.93 (*p* < 0.001), 0.88 (*p* < 0.001), and 0.91 (*p* < 0.001). Pearson correlations between age and BAD were −0.59 (*p* < 0.001), −0.37 (*p* < 0.001), and −0.50 (*p* < 0.001), respectively. The training process is visualised in Figure 3, and scatterplots of true versus predicted age (brain age) are displayed in Figure 4.

#### Centralised benchmark

Centralised training on the three open-source datasets took 3 h, 20 min, and 33 s. The model reached a minimum after 18 rounds and was stopped early after 38 rounds. The model achieved an overall test MAE of 7.02, a Pearson correlation (age and brain age) of 0.87 (*p* < 0.001), and a Pearson correlation (age and BAD) of −0.53 (*p* < 0.001). On CamCAN, IXI, and SALD, respectively, the model predicted brain age with a test MAE of 6.72, 6.78, and 7.70; a Pearson correlation (age and brain age) of 0.91 (*p* < 0.001), 0.86 (*p* < 0.001) and 0.86 (*p* < 0.001); and a Pearson correlation (age and BAD) of −0.71 (*p* < 0.001), −0.35 (*p* < 0.001), and −0.44 (*p* < 0.001). The overall results are displayed in Supplementary Figure 2 (loss figure) and Supplementary Figure 3 (test results scatterplot).

### Step 2: predicting SDMT

**Figure 5:**
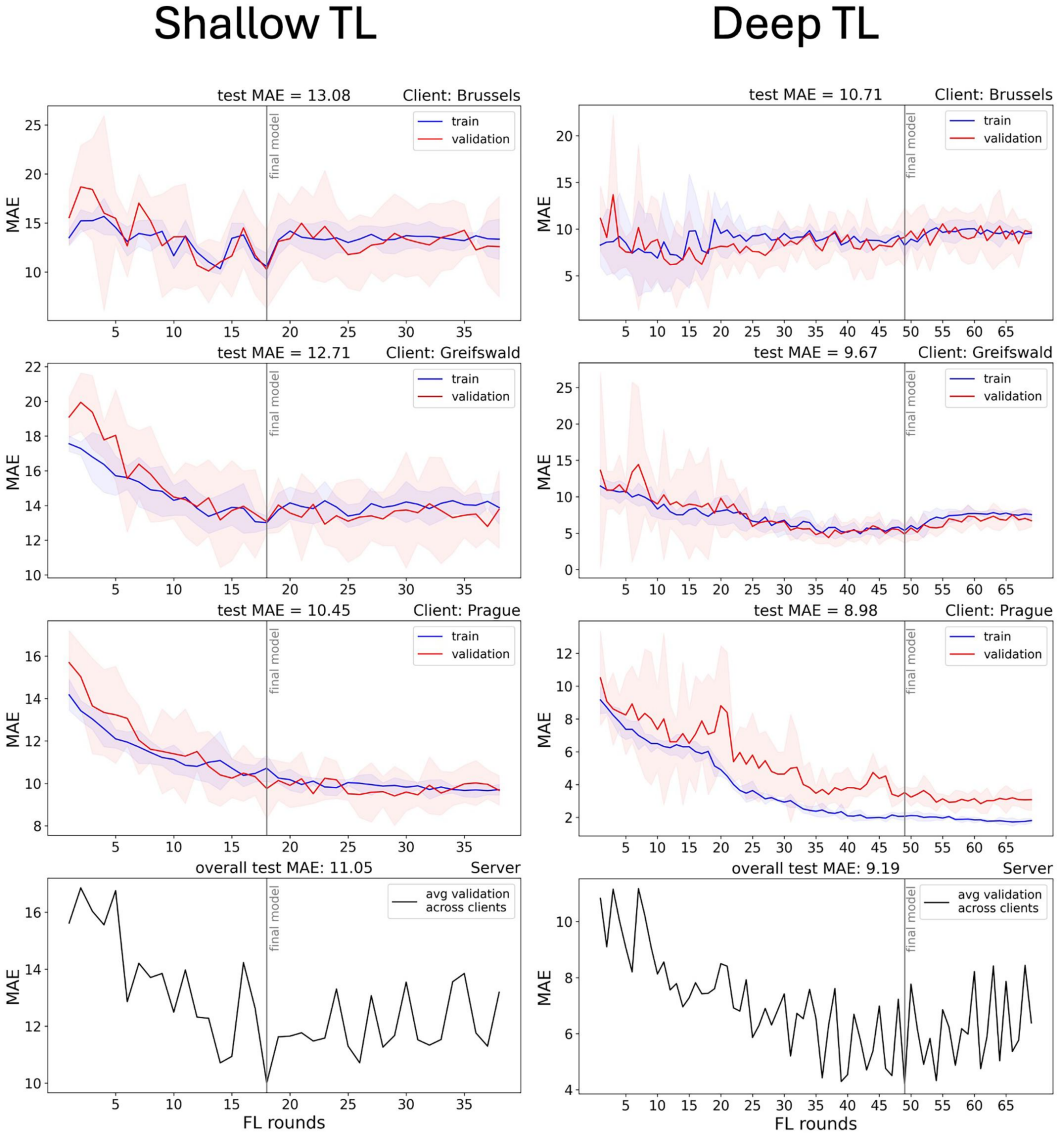
Loss figure of fine-tuning the brain age model to predict SDMT using shallow (left) and deep (right) transfer learning. FL, federated learning; MAE, mean absolute error; avg, average. The lower panels show the average validation MAE across the clients in the aggregation, calculated on the server side.

**Figure 6:**
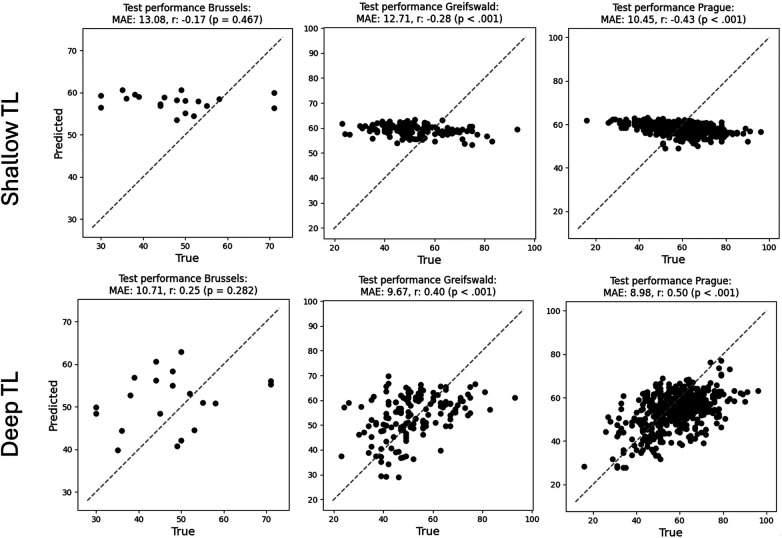
Scatterplots visualising test performance of the SDMT model trained with shallow TL (upper row) and deep TL (lower row).

Figure 5 illustrates the training process for the SDMT models, using shallow TL (left) and deep TL (right), starting from the centralised brain age model. Figure 6 shows the results of applying the final model to the test set of each centre.

Shallow TL took 3 h, 21 min, and 3 s. The model reached a minimum average validation MAE after 18 FL rounds and achieved an overall test MAE of 11.05 SDMT points. The per-client test MAEs were 13.08 (Brussels), 12.71 (Greifswald), and 10.45 (Prague), whereas the Pearson correlations were −0.17 (*p* = 0.467), −0.28 (*p* < .001), and −0.43 (*p* < .001), respectively.

For the deep TL model (right), training took 8 h, 4 min, and 35 s. The model reached a minimum at FL round 49 and achieved an overall test MAE of 9.19 SDMT points. The per-client test MAEs were 10.71 (Brussels), 9.67 (Greifswald), and 8.98 (Prague), whereas the Pearson correlations were 0.25 (*p* = 0.282), 0.40 (*p* < 0.001), and 0.50 (*p* < 0.001), respectively.

## Discussion

In this manuscript, we introduced FLightcase, a federated learning toolbox specifically designed to promote real-world, decentralised machine learning for brain scientists. We then demonstrated its real-world readiness by training models to predict SDMT from T1-weighted brain MRI images, using transfer learning from a brain age model. During brain age modelling, federated training outperformed centralised training. During transfer learning, a deep paradigm—where all network weights were trained—outperformed shallow learning—where only the last network layer was updated. The final model predicted SDMT with an average error of 9.19 points and performed especially well on the Greifswald and Prague datasets.

### Decentralised brain age modelling

The final federated brain age model predicted age from T1w MRI of healthy controls with an average error of 6.08 years across datasets. Strong correlations were observed on each client test dataset, indicating that federated learning successfully sensitised the model to individual differences in brain structure. Federated learning also outperformed centralised training with a decreased MAE of 0.94 years. Notably, in earlier experiments, where train and validation splits were not harmonised between federated and centralised experiments, the inverse was observed. An FL simulation study by Basodi et al. found similar performances of brain age models trained on decentralised and centralised datasets (12). In their study, besides a larger dataset of over 10,000 images from two sources, data from each source was distributed across six simulated centres, contrasting with our design of one source per centre. Overall, these results indicate that federated training can be a compelling alternative to a centralised training paradigm when data cannot be shared.

Our brain age models may suffer from low generalisability due to the nature of the open-source datasets, which contained both Caucasian (CamCAN and IXI) and Chinese (SALD) brains, known to differ in structure (48, 49). The model indeed had a higher test MAE in SALD (6.22) compared to CamCAN (5.91) and IXI (6.15). Although the test performance per sample was similar, the zigzagging pattern at the end of training (Figure 3) reveals the importance of client sampling; valleys coincided with IXI and SALD being included in the sample. This zigzagging pattern was especially clear in the last rounds (Figure 3). This highlights the importance of considering alternative client sampling schemes such as FedSampling (50). Lastly, the combined size of the decentralised dataset was 1,730 images, while the best model reported in a comprehensive review on brain age model performance (6) was trained by Peng et al. on 14.503 T1-weighted brain images (51). Enlarging our dataset would likely have improved brain age prediction performance and reduced discrepancies between centralised and federated training, such as observed in Basodi et al. (12).

Finally, our brain age models displayed the well-described bias in which brain age is inherently overestimated in younger individuals and underestimated in older individuals. This was evident from the negative correlations between BAD and age. Research often corrects for this bias (52), although the effectiveness of these methods for downstream tasks has been questioned by Zhang et al. (47). Although it is beyond the scope of this manuscript to further discuss brain age correction, we underline its importance when using the models for downstream tasks.

### Transfer learning to SDMT

Deep transfer learning from the centralised brain age model outperformed shallow transfer learning, with an overall MAE of 9.19 versus 11.05. This indicates that the latent feature representation for the brain age task was incompatible with the SDMT prediction task, requiring an update of the feature extractor part of the network.

Although deep TL substantially improved performance, the model had difficulty decoding SDMT performance on the Brussels dataset. While training and validation loss, as well as confidence intervals, gradually decreased in Greifswald and Prague, loss increased in the Brussels dataset. Not unlike federated brain age modelling (cfr. supra), this translates into heavy loss fluctuations on the server side, depending on which centre models are in the aggregation sample. Valleys coincided with the local models of Greifswald and Prague in the aggregation sample. We hypothesise two factors underlying this behaviour.

First, the Greifswald and Prague datasets were larger. The contributions of their local models in each round were therefore bigger than the Brussels local model, causing the model to learn less from the Brussels dataset. This is an inherent problem of the conventional FedAvg paradigm, which is why solutions for equality of local model updates have started to emerge. An example of this is q-FedAvg, which “reweights” the loss by a parameter q (53). The result is that client models with higher loss receive a higher weight in the aggregation.

Second, the behaviour nicely illustrates the non-IID problem in federated learning, meaning that we cannot assume that data points are drawn from the same underlying sample (54). This violates an important assumption in machine learning (55). Indeed, MS centres work with different equipment and workflows, which can be expected to increase across country borders. As illustrated in Table 1, different MRI scanners were used across different centres, and MS samples differed, most prominently in the SDMT ground truth distribution. This underscores the importance of data harmonisation and understanding between-centre differences, including test administration practices. It is therefore encouraging to observe the emergence of open-source federated harmonisation toolboxes like FedHarmony (65), which should be considered in future research for optimal model performance.

### The road ahead for real-world FL in neuroimaging

Federated learning offers a compelling alternative to centralised machine learning. It distributes computational load and addresses data sharing—one of the major impediments to international scientific collaborations (56). The promises for federated learning in the medical field are substantial, with some of the most notable breakthroughs emerging from cancer research. One of the first large-scale real-world FL demonstrations was reported in 2022 by Pati and colleagues (15). In a global federated learning setting involving 71 institutions, they updated a publicly available algorithm to detect glioblastoma boundaries and obtained a considerable gain in the validation Dice score across clients of about 25%. The authors stated the following: “It is the use of FL that successfully enabled (i) access to such an unprecedented dataset of the most common and fatal adult brain tumour, and (ii) meaningful ML training to ensure the generalisability of models across out-of-sample data.” Indeed, although Vo et al. confirmed that comparable results can be obtained in centralised contexts in ideal scenarios (57), the key benefit of FL is that it can still achieve these results when this is not the case. Similarly, technical advances are emerging that enable one to address data heterogeneity across institutions in a federated setting, such as tackling the non-IID issue by a combination of distributed gradient blending and proximity-aware client weighting (58).

Considering these medical FL breakthroughs, it is even more surprising that literature often ignores the very first step to achieving these results: how to build an FL network across clinical institutions. This practical step appears to be an assumption, as the literature focusses on challenges after a network has been established (59). In our experience, however, building a real-world FL network is the key roadblock between the compelling idea of federated learning and its practical realisation, and should be the key focus to usher in an era of real-world decentralised machine learning beyond simulation.

#### Collaboration

A first prerequisite for building an FL network is a trust among all partners. With malicious intent, source data could, for example, be shared between computers via VPN. The team should moreover be multidisciplinary, containing both medical experts and technical experts in information technology (IT) and data science. Real-world federated learning poses unique IT challenges in terms of communication and encryption, in addition to other existing technical challenges in deep learning research. On the other hand, medical expertise is required in pre-processing and interpreting the data, as well as understanding the model and its output.

#### Hardware

Besides sufficient storage, updating neural networks such as the DenseNet model (over 11 million parameters) requires processing units like a graphical (GPU) or tensor processing unit (TPU) on each client computer. In our network, all client computers were equipped with a GPU with 24GB of random access memory (RAM).

#### Software

There is a lack of FL toolboxes that are rigorously tested in real-world circumstances, with most designed as general frameworks rather than specifically tailored to medical data needs. We began designing FLightcase in early 2023, when real-world FL examples in neuroimaging had just started to emerge (15). Although some toolboxes such as Flower (13) and OpenFL (60) were available open source, we experienced difficulties in real-world deployment on our own network. We therefore set out to design a framework relying on a simple but stable connection between computers, focussing on the core needs of our methodology, such as compliance with BIDS, support for loading anatomical brain images, and focussing on the original and most popular FL algorithm, FedAvg. Since then, toolboxes such as Flower have further developed into communities with peer support. Awareness about the difficulty of real-world FL is increasing, reflected by companies facilitating deployment such as ScaleOut, and efforts are underway to tailor toolboxes to the specific needs of different modelling domains. Almost a decade after the pioneering work of Plis and colleagues (61) on standardised analysis of decentralised neuroimaging data, FL software is approaching a technology readiness level where centralised workflows can be seamlessly converted to federated ones. For FLightcase in particular, we have reconsidered the basic communication layer, allowing file sharing by uploading to and downloading from a Flask web server.

#### Connectivity and remote access

All computers in our network were connected to locally available internet providers. This introduces new centre-specific difficulties pertaining to local security settings. Blocking web addresses such as TeamViewer, for example, complicated remote access. Combined with limited scalability due to a maximum number of remote computers in TeamViewer, we switched to tmate (cfr. methods). The drawback of this approach is the absence of a graphical interface; remote computers are operated via command line.

#### Financial

Ensuring the aforementioned requirements comes with a significant cost. In particular, the hardware for each client with expensive GPUs imposes additional financial restraints, limiting the feasibility of setting up a network.

### Limitations

Federated averaging (FedAvg) was the original aggregation algorithm when federated learning was introduced by H. Brendan McMahan and colleagues in 2016. Ten years after its introduction, the algorithm remains popular and attractive for its simplicity, which resonates with this manuscript's core message of facilitating real-world federated learning. Over the years, however, FedAvg has been criticised for reduced performance under non-IID circumstances (62). Newer algorithms—such as the “federated learning approach based on a greedy algorithm” [FedGA, (62)]—show promise in overcoming this limitation.

### Final considerations

The goal of any medical AI endeavour is to deliver models that can be used in a clinical setting. Simultaneously, we strive for generalisability of models. In a multi-centre federated learning setting, models may perform worse in a single-centre context as they attempt to find the “middle ground.” Going back to the core objective of delivering clinically ready AI tools, the question can thus be raised whether overfitting on single-centre data is truly problematic. Moreover, FL risks encoding centre-specific bias pertaining to, for example, test administration and MR scanner properties. The central challenge is therefore how to best use large multi-centre data.

In line with the current trend of building so-called “foundation models” (63), the way forward may be to use a transfer learning paradigm where multi-centre data are first used to create a strong foundation model that encodes as much centre-independent information as possible. Transfer learning can then be employed to fine-tune the model on single-centre datasets. In this manuscript, we attempted to do so by creating a “brain age foundational model.” Although we achieved fair performance on the downstream SDMT task, we consider an “SDMT foundational model” possible by investing in real-world FL. By offering scientists a starting point to engage in real-world FL, we hope our manuscript facilitates the creation of strong cognitive screening models across diverse clinical settings.

## Data Availability

The data analysed in this study are subject to the following licenses/restrictions: data underlying the real-world and simulated brain age experiments are open source available. Other datasets are accessible upon reasonable request. Requests to access these datasets should be directed to stijn.denissen@vub.be.
